# Association between the PPP3CC gene, coding for the calcineurin gamma catalytic subunit, and bipolar disorder

**DOI:** 10.1186/1744-9081-4-2

**Published:** 2008-01-17

**Authors:** Flavie Mathieu, Stéphanie Miot, Bruno Etain, Marie-Anne El Khoury, Fabien Chevalier, Frank Bellivier, Marion Leboyer, Bruno Giros, Eleni T Tzavara

**Affiliations:** 1 INSERM U-513, Faculté de Médecine, 8 rue de Général Sarrail, 94000, Créteil, France; 2 AP-HP, Albert Chenevier and Henri Mondor Hospitals, Department of Psychiatry, 94000, Créteil, France

## Abstract

**Background:**

Calcineurin is a neuron-enriched phosphatase that regulates synaptic plasticity and neuronal adaptation. Activation of calcineurin, overall, antagonizes the effects of the cyclic AMP activated protein/kinase A. Thus, kinase/phosphatase dynamic balance seems to be critical for transition to long-term cellular responses in neurons, and disruption of this equilibrium should induce behavioral impairments in animal models. Genetic animal models, as well as post-mortem studies in humans have implicated calcineurin dependent calcium and cyclic AMP regulated phosphorylation/dephosphorylation in both affective responses and psychosis. Recently, genetic association between schizophrenia and genetic variation of the human calcineurin A gamma subunit gene (PPP3CC) has been reported.

**Methods:**

Based on the assumption of the common underlying genetic factor in schizophrenia and bipolar affective disorder (BPAD), we performed association analysis of CC33 and CCS3 polymorphisms of the PPP3CC gene reported to be associated with schizophrenia in a French sample of 115 BPAD patients and 97 healthy controls.

**Results:**

Carrying 'CT' or 'TT' genotypes of the PPP3CC-CC33 polymorphism increased risk to develop BPAD comparing to carry 'CC' genotype (OR = 1.8 [1.01–3.0]; p = 0.05). For the PPP3CC-CCS3 polymorphism, 'AG' or 'GG' carriers have an increased risk to develop BPAD than 'AA' carriers (OR = 2.8 [1.5–5.2]). The CC33 and CCS3 polymorphisms were observed in significant linkage disequilibrium (D' = 0.91, r^2 ^= 0.72). Haplotype frequencies were significantly different in BPAD patients than in controls (p = 0.03), with a significant over-transmission of the 'TG' haplotype in BPAD patients (p = 0.001).

Conclusion:

We suggest that the PPP3CC gene might be a susceptibility gene for BPAD, in accordance with current neurobiological hypotheses that implicate dysregulation of signal-transduction pathways, such as those regulated by calcineurin, in the etiology of affective disorders.

## Background

Calcineurin (CN; also known as Ca2+/protein phosphatase 2B) is a neuron enriched calcium/calmodulin stimulated serine/threonine phosphatase composed of a regulatory subunit (CNB) and a catalytic subunit (CNA). There are three mammalian calcineurin catalytic isoforms (CNA α, β and γ) and two regulatory isoforms (CNB1 and CNB2). Acting as calcium-sensor, calcium-G protein signaling integrator, and regulator of calcium homeostasis, calcineurin shapes calcium and cyclic AMP dependent processes: synaptic activity, receptor desensitization, cell survival, neuroplasticity, cellular resilience [1]. Calcineurin activation, overall, opposes cyclic AMP/PKA actions, and the fine-tuning of this kinase/phosphatase balance is critical for neuronal adaptation, transition to long-term responses and higher functions including sensitivity to aversive stimuli, fear conditioning, and importantly learning and memory [1].

Recently, different lines of evidence have suggested that calcineurin could be a possible risk factor of schizophrenia. Most importantly, Gerber and collaborators [2] reported the association of schizophrenia with genetic variation of the PPP3CC gene, which encodes the CNA-γ-subunit in a Caucasian population. The PPP3CC catalytic subunit of calcineurin is located at 8p21, a highly replicated schizophrenia susceptibility locus, and variants within the gene are associated with schizophrenia. This association is not seen in a Japanese sample [3]. However, studies with transgenic animal models [4,5] show that forebrain specific CNB knockout mice display behavioral abnormalities reminiscent of both schizophrenia, such as increased locomotion, decreased social interaction and deficits in working memory and sensorimotor gating. In addition pharmacological [6,7] studies in animals, as well as post-mortem findings in schizophrenic patients [[8] but see [9]] show that expression of calcineurin is altered upon treatment with anti-psychotics and expression of the PPP3CC and other calcineurin subunits is decreased in the hippocampus of schizophrenic patients further implicating calcineurin in neuropsychiatric disorders, such as schizophrenia [10].

Alterations in calcineurin signaling may also be involved in bipolar affective disorder (BPAD). Many of the phenotypes of the CNB knock out mouse are also relevant to BPAD, calcineurin subunits are down-regulated in the pre-frontal cortex of BPAD patients and other genes involved in calcineurin signaling, DARPP-32 (dopamine and cyclic AMP regulated phosphoprotein of Mr 32,000), CDK5 (cyclin dependent kinase5), GSK3 (glycogen kinase sytnthase 3) and the calcineurin activated adenylyl cyclase 9, have been associated with BPAD [11,12]. Finally, 8p21 has also been identified as a highly significant susceptibility locus for a psychotic form of BPAD [13].

## Methods

We hypothesized that variants in the PPP3CC catalytic subunit might also predispose to BPAD. We investigated the association between PPP3CC and bipolar-affective-disorder (BPAD) in a Caucasian French population of 115 BPAD patients and 97 healthy controls. BPAD patients were recruited at two French University-affiliated Psychiatric Departments (one in Paris and one in Bordeaux). Inclusion criteria comprised: BPAD I or II diagnosis (according to DSM-IV criteria); Caucasian origin with at least three out of four grandparents being French; being at least 18 years old; being euthymic (having a MADRS (Montgomery-Asberg Depressive Rating Scale) score of fifteen or less and a MAS (Bech and Raphaelsen Mania Rating Scale) score of five or less) at inclusion. The control group comprised randomly selected blood donors having no personal or family history of any psychiatric disorder. Written informed consent was obtained from all participants. A trained psychiatrist using the French version of the Diagnostic Interview for Genetic Studies (DIGS) providing lifetime DSM-IV axis I diagnoses interviewed patients and control subjects. Male/female ratio was 0.72 in BPAD patients and 1.68 in controls. Based on the PPP3CC/schizophrenia association study [2], we genotyped the two intronic single-nucleotide polymorphisms (SNPs) showing strongest evidence for linkage (CC33T/C and CCS3A/G). We also genotyped the coding sequence mutation identified [2] on PPP3CC exon 5, resulting in a nonconservative change in the amino acid sequence of the protein from a charged arginine at position 163 to a neutral glutamine (CC-5).

## Results and discussion

We identified the CC-5 mutation in 3 patients and in 0 controls, a frequency comparable to that of the PPP3CC/schizophrenia study (i.e. 3 of 210 tested patients and 0 of 75 Caucasian controls; [2]). For CC33 and CCS3, Hardy-Weinberg-equilibrium was tested separately in cases and controls. We also estimated linkage disequilibrium values D' and r^2 ^[14,15]. We tested for disease-haplotype association by likelihood-based approaches using the EH-plus software [16]. No deviation from Hardy-Weinberg-equilibrium was observed for CC33 and CCS3, in both groups. Association between BPAD and CC33 genotype was borderline significant (p = 0.07), with an higher risk to develop bipolar disorder for 'CT' or 'TT' carriers versus 'CC' carriers (OR = 1.8 [1.01–3.0]; p = 0.05) and a significant allelic association was observed with the 'T'-allele of CC33 (p = 0.05, see Table 1 for details). Our results show also a significant association between BPAD and CCS3 (p = 0.003), with an higher risk to develop bipolar disorder for 'AG' or 'GG' carriers versus 'AA' carriers (OR = 2.8 [1.5–5.2]) and a significant allelic association between the 'G'-allele of CCS3 and BPAD was observed (p = 0.002, see table 1 for details). The CC33 and CCS3 polymorphisms were observed in significant linkage disequilibrium (D' = 0.91, r^2 ^= 0.72). Haplotype frequencies were significantly different in BPAD patients than in controls (Likelihood ratio test: Chi^2 ^(3 ddf) = 9.08, p = 0.03), and a significant over-transmission of the PPP3CC haplotype 'TG' (p = 0.001) was observed for bipolar patients. HapMap LD data for the PPP3CC gene support the association being due to a variant in PPP3CC and not a nearby gene.

**Table 1:** Genotypic, Allelic and Haplotype association study of PPP3CC -CC33 and -CCS3 polymorphisms

	**Allele Count**	**Cases**	**Controls**	**OR [CI_95%]**	**p-value**
**CC33**	CC	39	46	(CT+TT) versus CC 1.8 [1.01–3.0]	0.05
	CT	52	36		
	TT	24	15		
					
	f(C)	0.57	0.66	-	
	f(T)	0.43	0.34	1.5 [1.01–2.2]	0.05

**CCS3**	**Allele frequencies**	**Cases**	**Controls**	**OR [CI_95%]**	**p-value**

	AA	22	39	(GG+AG) vs AA 2.8 [1.5–5.2]	0.001
	AG	60	41		
	GG	27	14		
					
	f(A)	0.48	0.63	-	
	f(G)	0.52	0.37	1.9 [1.3–2.8]	0.002

**CC33/CCS3**	**Haplotype frequencies**	**Cases**	**Controls**	**Chi-Square**	**p-value**

	CA	0.47	0.61	TG versus (C2+CG+TA) 16.2	0.001
	CG	0.09	0.05		
	TA	-	0.02		
	TG	0.44	0.32		

In order to test an association between psychotic symptoms and PPP3CC polymorphisms, we have compared genotypic repartition in psychotic (N = 59) versus non-psychotic (N = 56) patients for both CC33 and CCS3 polymorphism. No difference was observed. Moreover, psychotic and non-psychotic patient samples shown similar association when compared to controls, suggesting that these variants are implicated in bipolar disorder per se and are not specific of psychotic symptoms.

As male/female ratio was significantly different between patients and controls, we have performed genotypic and haplotypic analyses stratified on gender. Similar results were observed: a higher risk to develop bipolar disorder for 'CT' or 'TT' carriers for CC33: OR = 1.45 [0.67–3.15] for males and females OR = 2.54 [1.10–5.91]; association between bipolar disorder and CC33 being significant only for females (p = 0.03). For CCS3 polymorphism, 'AG' or 'GG' carriers have a higher risk than 'AA' carriers to develop disease, (males OR = 1.29 [0.92–1.83] and females OR = 2.57 [1.57–4.21]). However, association between bipolar disorder and CC33 is significant only for females (p < 0.001). Also association of CC33 haplotypes was significant in females (Chi^2 ^(3 df) = 50.6; p < 0.001) and not significant in males (Chi^2 ^(3 df) = 4.4; p = 0.22). An over transmission of 'TG' haplotype was observed for bipolar males and female patients.

Our results suggest that the PPP3CC gene may contribute to the etiology of BPAD. This is in accordance with preclinical work that relates calcineurin related signaling pathways to the pathophysiology of affective and cognitive disorders. Specifically it was shown that phosphatase activity/amount could predict whether a signaling system will operate in a proportional-response-mode, or as an all-or-none-switch [17] susceptible to generate disproportional network responses. This might be relevant to the bidirectional behavioral dysfunction (depressed or agitated mood) characterizing BPAD. Furthermore, as stated above, preclinical studies have implicated calcineurin in cognitive function as well as in affective responsiveness [1,5,18]. Our present findings suggest that PPP3CC might be a shared susceptibility gene for schizophrenia and BPAD. Calcineurin dysregulation, therefore, might be associated with transnosographic phenotypical aspects, such as affective and cognitive pathology, seen in both disorders.

## Conclusion

We suggest that the PPP3CC gene might be a susceptibility gene for BPAD, in accordance with the current neurobiological hypothesis that implicates dysregulation of signal-transduction pathways, such as those regulated by calcineurin, in the etiology of affective disorders. We would like however to point out some limitations related to our approach. One must be aware that case/control association studies often lead to non-replicable results. In this respect our population is very small and ethnographic bias cannot be excluded. In conclusion this is a case/control report with limited power that reports for the first time an association between calcineurin and BPAD. Further investigations with larger samples are warranted to confirm this result.

## Abbreviations

BPAD: Bipolar affective disorder; CN: Calcineurin.

## Competing interests

The author(s) declare that they have no competing interests.

## Authors' contributions

FM and SM contributed equally to this work. FM participated in the study design and coordination, performed statistical analysis and wrote the manuscript, SM carried out the molecular genetic studies, participated in the sequence alignment, collacted and analyzed the data and drafted the manuscript, BE and FB paricipated in patient recruitment, interview and evaluation, M-A. EK and FC participated in the genotyping, ML is the principal investigator of the clinical protocols, BG is the principal investigator of the molecular protocols, ETT participated in study design and coordination, participated in patient interview and genotyping, collected data and wrote the manuscript.
